# Supplementary material for: Physiological Ovarian Aging Is Associated with Altered Expression of Post-Translational Modifications in Mice
Source: Int J Mol Sci. 2021 Dec 21;23(1):2. doi: 10.3390/ijms23010002 (PMC8744712; doi:10.3390/ijms23010002)
Supplement: Supplementary file 1 [file ijms-23-00002-s001.zip › ijms-1503716-supplementary.pdf]

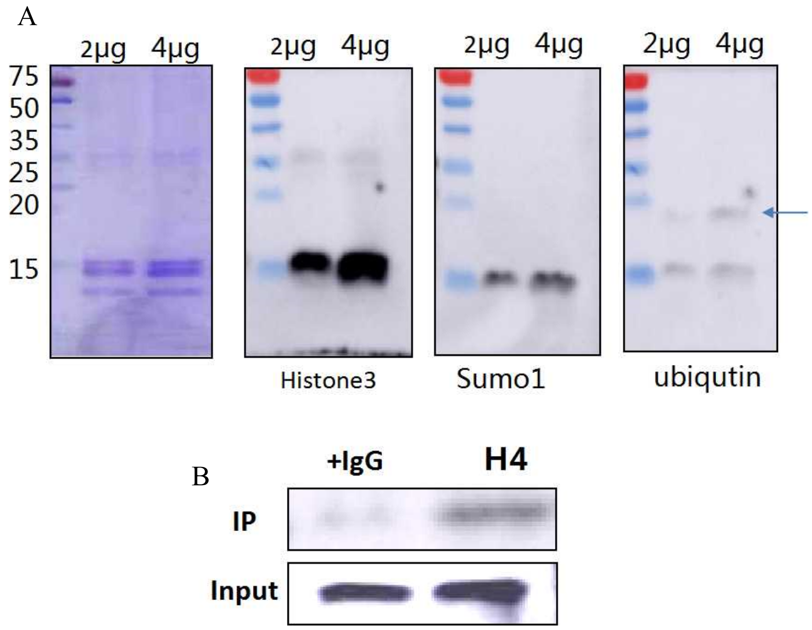

Figure S1. The modification changes of SUMO1 and Ubiquitin in histone by immunoprecipitation. A: the results suggested that the level of SUMO1 do not occur during ovarian histone in mice. B: the expression of Ubiquitin were observed in Histone4.

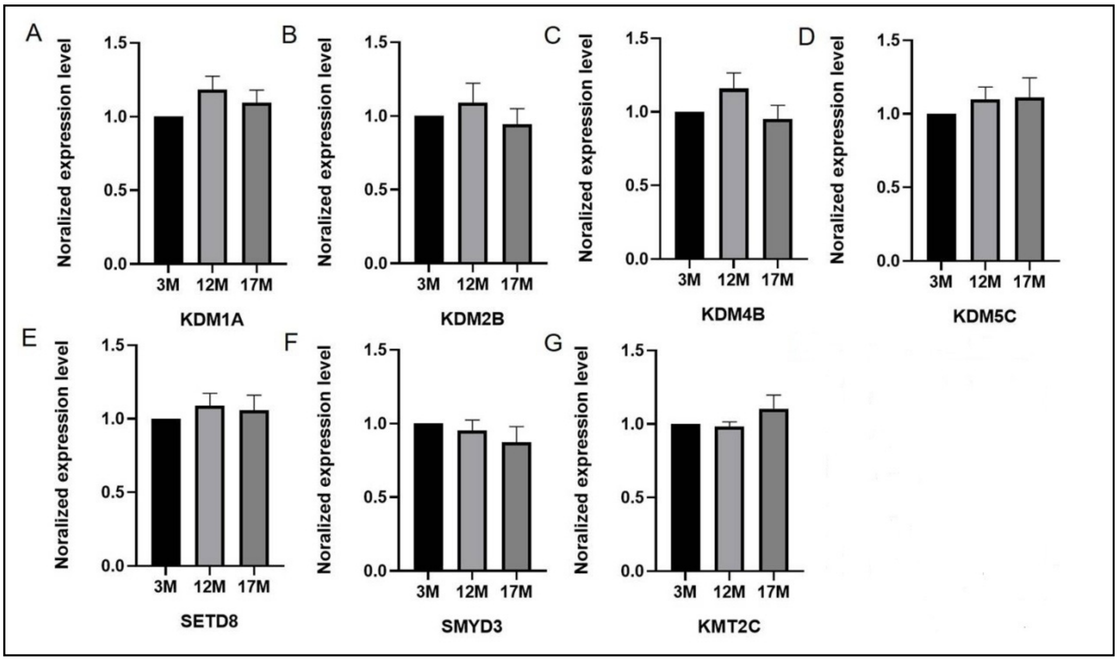

Figure S2. The expression of related regulatory enzymes were examined by quantitative real-time PCR using the $2^{-\triangle\triangle Ct}$ method. KDM1A, Lysine Demethylase 1A; KDM2B, Lysine Demethylase 2B; KDM4B, Lysine Demethylase 4B; KDM5C, Lysine Demethylase 5C; SETD8, lysine methyltransferase 8; SMYD3, SET And MYND Domain Containing 3; KMT2C, Lysine methyltransferase 2C. Bar: mean ± SEM.

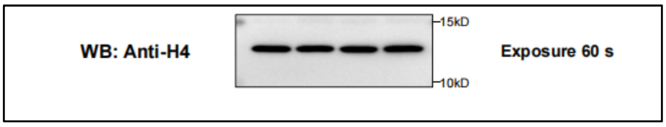

Figure S3. Western blotting with anti-H4 antibody.

Table S1. Primers used for quantitative-PCR

Gene	Primer Sequence(5'-3')	Sequence number	Gene	Primer Sequence(5'-3')	Sequence number
MVH	F:GTGTATTATTGTAGCACCAACTCG<br>R:CACCCTTGTACTATCTGTCGAACT	NM:010029.2	KDM1A	F:CGATACTGTGCTTGTCCACCGA<br>R:CCAAGCCAGAAACACCTGAACC	NM_133872
AMH	F:CCGCTATTTGGTGCTAACCGTG<br>R:AAGGCTTGCAGCTGATCGATGC	NM_007445.3	KDM2B	F:GGTGTGGTCAACGATGAGCTTC<br>R:CGGTTCATCTTCTGCTCCTTGAG	NM_001005866
TIP60	F:TGAGCGTGAAGGACATCAGTGG<br>R:TTAAGTCCAGCCGCTCGTGAGT	NM_001199247	KDM4B	F:TGGTGTCCATCACCTGCCTCAA<br>R:CATTGCGGTTCTTGGTGATGACG	NM_172132
P300	F:GTGATGACCCTTCCCAACCTCA<br>R:CTCGTGGTGAAGGACACAGATC	NM_177821	KDM5C	F:AGCTGAGTCCTTTGACACCTGG<br>R:TTCGCTCACGAGCCTCAGATTC	NM_013668
SAE1	F:GTAAGCCAAGGAGTGGAAGATGG<br>R:CAGTCCACTTCTAGCGCCTCTT	NM_001285891	SETD8	F:TCACTGATGCCAAGAAGCGGGA<br>R:GCGGTTCGTTTCTTGAGTGGCA	NM_001310723
PRDM9	F:AGGGAAGAAACTGCTATGAG<br>R:AAGATTTCAGTGCGATGAT	NM_020227	SMYD3	F:CTACCTGGACATGCTGATGACC<br>R:CAGCGACTCTTGAACCTCCTTC	NM_027188
KMT5B	F:AGATGCAGACACCACTCAGGAAA<br>R:GGTGAGGTAGAGTTGGAAGCAG	NM_001167884	KMT2C	F:CCTATCCTCCAGAGGTTGCTGG<br>R:TTTGCTGAGGCACATGGAAGCG	NM_001081383
KMT5C	F:TGCCTGAGAGTGGCTTCACCAT<br>R:TCCTCTTCACGCAGCTCTGCAA	NM_146177	GAPDH	F:ACGTGCCGCCTGGAGAAAC<br>R:GTCCTCAGTGTAGCCCAAGATGC	XM_001476707.<br>3

Table S2. IACUC study protocol

Animal Use Permit	
**License No. :**	**SYXK(gan)-2015-0001**
**Name of the entity:**	**Nanchang University**
**Legal representative:**	**Changbing Zhou**
**Facility Address:**	**Medical Laboratory Animal Center, Nanchang University, No. 71 East Yangming Road, Nanchang city**
**Application:**	**barrier environment**

Table S3. specify vendor catalog # details of all the antibody used in this study

Product code	Primary antibody	dilution rate	Product code	Primary antibody	dilution rate
PTM-1004 Lot: Z390I813P1	Anti-histone H4 rabbit pAb	1:1000	PTM-701; Lot: 10201114G705	Anti-phosphotyrosine Antibody	1:1000
PTM-101; Lot: 10167J809	Anti-acetyllysine Antibody	1:1000	PTM-752 ; Lot: 2286515FB28	Anti-nitro-Tyrosine Antibody	1:1000
PTM-201; Lot: Z454J312P5	Anti-propionyllysine antibody	1:1000	PTM-762: Lot: 1314381916J916	Anti-benzoyllysine Antibody	1:300
PTM-301; Lot: ZF419J715P2	Anti-butyryllysine Antibody	1: 500	PTM-802; Lot:13592312J110	Anti-2-hydroxyisobutyryllysine Antibody	1:750
PTM-419; Lot: 105032317G009	Anti-succinyllysine Antibody	1:1000	PTM-902; Lot:2305341HA07	Anti-malonyllysine Antibody	1:2000
PTM-502; Lot:1037267J418	Anti-crotonyllysine Antibody	1:2000	PTM-952; Lot: 1G0252543	Anti-O-GlcNAc Antibody	1:1000
PTM-601; Lot: ZD151G017P4	Anti-tri-methyllysine Antibody	1:1000	PTM-1107; Lot: 21671155J902	Anti-ubiqutin Antibody	1:2000
PTM-606; Lot: ZD068GB14P56	Anti-di-methyllysine Antibody	1:1000	PTM-1110; Lot: 100786G16	Anti-sumo1 mouse mAb	1:1000
PTM-1151; Lot: ZC061IA15P0	Anti-glutaryllysine	1:1000	PTM-1201; Lot:ZD527J816P0	Anti-β-hydroxybutyryllysine rabbit pAb	1:1000
PTM-1401: Lot:Z264J028P0	Anti-lactyllysine Antibody	1:2000	protentech Cat No: 60004-I-Ig	Anti-GAPDH Antibody	1:5000

Table S4. Language modification voucher

Proof-Reading-Service.com

PhD theses, journal papers, books and other professional documents

Proof-Reading-Service.com Ltd, Devonshire Business Centre, Works Road, Letchworth Garden City, Hertfordshire, SG6 1GJ, United Kingdom
Office phone: +44(0)20 31 500 431
E-mail: enquiries@proof-reading-service.com
Internet: http://www.proof-reading-service.com
VAT registration number: 911 4788 21
Company registration number: 8391405

07 September 2021

To whom it may concern,

RE: Proof-Reading-Service.com Editorial Certification

This is to confirm that the document described below has been submitted to Proof-Reading-Service.com for editing and proofreading.

We certify that the editor has corrected the document, ensured consistency of the spelling, grammar and punctuation, and checked the format of the sub-headings, bibliographical references, tables, figures etc. The editor has further checked that the document is formatted according to the style guide supplied by the author. If no style guide was supplied, the editor has corrected the references in accordance with the style that appeared to be prevalent in the document and imposed internal consistency, at least, on the format.

It is up to the author to accept, reject or respond to any changes, corrections, suggestions and recommendations made by the editor. This often involves the need to add or complete bibliographical references and respond to any comments made by the editor, in particular regarding clarification of the text or the need for further information or explanation.

We are one of the largest proofreading and editing services worldwide for research documents, covering all academic areas including Engineering, Medicine, Physical and Biological Sciences, Social Sciences, Economics, Law, Management and the Humanities. All our editors are native English speakers and educated at least to Master's degree level (many hold a PhD) with extensive university and scientific editorial experience.

**Document title:**	**Physiological ovarian aging is associated with altered expression of post-translational modifications in mice**
**Author(s):**	**Jia Li**
**Format:**	**British English**
**Style guide:**	**Theriogenology at https://www.elsevier.com/journals/theriogenology/0093-691X/guide-for-authors**
